# Meningococcal Disease in US Military Personnel before and After Adoption of Conjugate Vaccine

**DOI:** 10.3201/eid2102.141037

**Published:** 2015-02

**Authors:** Michael P. Broderick, Christopher Phillips, Dennis Faix

**Affiliations:** Naval Health Research Center, San Diego, California, USA

**Keywords:** US military, meningococcal disease, vaccine, bacteria

**To the Editor:** Meningococcal disease in US military personnel is controlled by vaccines, the first of which was developed by the US Army (*1*–*5*). In 1985, the quadrivalent polysaccharide vaccine (MPSV-4) was implemented as the military standard. It was replaced during 2006–2008 by the quadrivalent conjugate vaccine (MCV-4). Every person entering US military service is required to receive this vaccine.

Meningococcal disease incidence in active-duty US military personnel, historically far above that in the general population (*6*), has decreased >90% since the early 1970s, when the first vaccine was introduced (*7*). Over the last 5 years, incidences in the military and US general populations have become equivalent (*8*). Here we update previously published data (*8*) from the Naval Health Research Center’s Laboratory-based Meningococcal Disease Surveillance Program of US military personnel. Data-gathering methods and laboratory analyses of samples from personnel suspected of having meningococcal disease have been previously described (*8*). Incidences were compared by using the New York State Department of Public Health Assessment Indicator based on the methods of Breslow and Day (*9*).

During 2006–2013 in US military personnel, only 1 of the 28 meningococcal disease cases for which serogroup data are available was not serogroups C or B (8 cases each) or Y (11 cases). During that period, incidence in US military personnel of 0.271 cases per 100,000 person-years did not differ significantly (p>0.05) from that of 0.238 in the 2006–2012 age-matched US general population (persons 17–64 years of age) (Centers for Disease Control and Prevention [CDC], unpub. data). During 2010–2013, meningococcal disease incidence in military personnel was 0.174 cases per 100,000 person-years, compared with 0.194 in the age-matched 2010–2012 US population. Among military personnel, only 1 case each occurred in 2011 (serogroup Y) and 2012 (serogroup B), and 3 occurred in 2013 (1 each of serogroups B, C, and Y).

To measure the relative success of the 2 vaccines, we compared incidence among military personnel who had received MPSV-4 with that of personnel who had received MCV-4. In 2006, MCV-4 was introduced to new recruits. The proportion of military personnel who had received MCV-4, rather than MPSV-4, increased from 6% of the military population (63,000 persons) in 2006 to 64% (930,000) in 2013. By 2013, a total of 99% of new vaccinations were of MCV-4. Overall incidence in personnel receiving MCV-4 was 0.298 cases per 100,000 person-years during 2006–2013, which was lower, although not significantly lower (p>0.05), than 0.410 cases per 100,000 person-years in MPSV-4 recipients during 2000–2013.

However, because neither vaccine covers serogroup B, excluding serogroup B cases in the vaccine-related incidence calculations might be more appropriate. Incidence in MCV-4–vaccinated personnel during 2006–2013, excluding serogroup B cases, was 0.183. Specific serogroup data are not available for 2000–2005, so to calculate non–serogroup B incidence during this period, we estimated the proportion of serogroup B cases by examining a range of estimates of serogroup B proportions derived from the true proportions in all 6-year periods during 1995–2012 in the US general population (range 21%–35%; 35% during 2000–2005) (CDC, unpub. data) and during 2006–2013 in US military personnel (range 22%–28%). Adopting (from our estimated range of serogroup B proportions) 21% as the percentage that would have made the MPSV-4–related incidence the highest, MPSV-4–related incidence (i.e., excluding serogroup B cases) during 2000–2013 would have been 0.307, which did not differ significantly from incidence of MCV-4 non-serogroup B cases (p>0.05). (Using higher percentages would have pushed the MPSV-4 estimate even closer to the MCV-4 incidence.) The Figure shows pooled incidence for 2000–2013.

**Figure:**
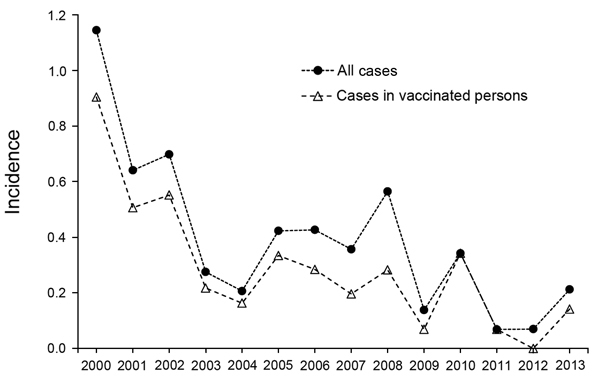
Meningococcal disease incidence per 100,000 person-years in US military personnel, 2000–2013. Incidence in vaccinated personnel shown assumes that 21% of cases during 2000–2005 were caused by *Neisseria meningitis* sergroup B.

Results of these comparisons are subject to several limitations. First, because the relative proportions of the 2 vaccines changed, a differential effect of herd immunity caused by one or the other could have differentially suppressed rates. Second, along with the decrease in the MPSV-4 population, the average time from vaccination increased relative to the period in which MPSV-4 was given, concomitant with decreasing immunogenicity. Any elevated incidence in the MPSV-4–vaccinated population since 2006 could be associated with time since vaccination. Third, the same factors involved in the decline in incidence in the US general population that began in ≈2001 might be at play in the military, confounding the vaccine effects. Fourth, as the rate of vaccine coverage in the US population increased, a higher proportion of recruits might have entered the military already vaccinated; thus, their military vaccination was essentially a booster.

Meningococcal disease incidence decreased during 2000–2013. Our data suggest that cases in MCV-4–vaccinated personnel are similar to those in MPSV-4–vaccinated personnel, regardless of whether the incidence calculation includes cases caused by serogroup B (non–vaccine covered). More extensive study is needed to confirm the relative effects of the vaccines (*10*). Serogroup B accounted for 5 of the 8 cases during 2012–September 2014), and prevention of disease caused by this serotype remains a challenge.
